# Understanding the Determination of Meat Quality Using Biochemical Characteristics of the Muscle: Stress at Slaughter and Other Missing Keys

**DOI:** 10.3390/foods10010084

**Published:** 2021-01-04

**Authors:** E. M. Claudia Terlouw, Brigitte Picard, Véronique Deiss, Cécile Berri, Jean-François Hocquette, Bénédicte Lebret, Florence Lefèvre, Ruth Hamill, Mohammed Gagaoua

**Affiliations:** 1 UMR Herbivores, INRAE, VetAgro Sup, Theix, 63122 Saint-Genès Champanelle, France; brigitte.picard@inrae.fr (B.P.); veronique.deiss@inrae.fr (V.D.); jean-francois.hocquette@inrae.fr (J.-F.H.); 2 INRAE, Université de Tours, BOA, 37380 Nouzilly, France; cecile.berri@inrae.fr; 3 UMR Physiologie, Environnement et Génétique pour l’Animal et les Systèmes d’Élevage, INRAE, AgroCampus Ouest, 35590 Saint-Gilles, France; benedicte.lebret@inrae.fr; 4 Laboratoire de Physiologie et Génomique des Poissons, INRAE, 35042 Rennes, France; florence.lefevre@inrae.fr; 5 Teagasc Ashtown Food Research Centre, Food Quality and Sensory Science Department, Ashtown, Dublin 15, Ireland; Ruth.Hamill@teagasc.ie (R.H.); gmber2001@yahoo.fr (M.G.)

**Keywords:** slaughter, stress, meat quality, behavior, physiology, postmortem muscle metabolism, biochemistry, proteomics, modeling

## Abstract

Despite increasingly detailed knowledge of the biochemical processes involved in the determination of meat quality traits, robust models, using biochemical characteristics of the muscle to predict future meat quality, lack. The neglecting of various aspects of the model paradigm may explain this. First, preslaughter stress has a major impact on meat quality and varies according to slaughter context and individuals. Yet, it is rarely taken into account in meat quality models. Second, phenotypic similarity does not imply similarity in the underlying biological causes, and several models may be needed to explain a given phenotype. Finally, the implications of the complexity of biological systems are discussed: a homeostatic equilibrium can be reached in countless ways, involving thousands of interacting processes and molecules at different levels of the organism, changing over time and differing between animals. Consequently, even a robust model may explain a significant part, but not all of the variability between individuals.

## 1. Introduction

The many studies aiming to elucidate the biochemical events explaining the instrumental and sensory properties of meat have produced considerable insights into the role of metabolic, proteolytic, apoptotic and oxidative processes underlying meat characteristics [1]. The various reports of significant statistical models explaining variations in meat quality reveal the relevance of these processes and their interactions [2,3,4]. Although they are statistically referred to as predictive models, these models are currently post-hoc descriptive models, the predictive value of which has rarely been confirmed subsequently with independent samples. Despite our growing knowledge of the biochemical pathways involved in the development of the phenotypic characteristics of meat, methods for the successful prediction of meat quality from observable phenomena remain elusive [5,6]. Most models take into account rearing and animal factors, as well as muscle characteristics [7,8,9]. However, one important factor is generally ignored: the preslaughter stress status of the animal, often simply because such information was not measured. Another major difficulty is that similar phenotypic outcomes can generally have multiple, interrelated causes. In other words, animals, muscles or meat presenting the same particular phenotype may do so for at least partly different underlying biological mechanisms. For example, meat may be light-colored for reasons related to pH or fiber composition or a combination of these factors. Consequently, it is not surprising that models developed with only part of the relevant information can only explain a subset of the variability between individuals and vary between experiments. 

The first part of the present paper illustrates the importance of considering the stress characteristics of the animal in models aiming to predict or explain variations in meat quality traits. We present examples of the potentially large effects of preslaughter stress status of the animal on meat quality and discuss the possible underlying mechanisms as well as the many gaps in our knowledge. The second part illustrates how the existence of multiple, interrelated causes underlying phenotypical characteristics—a general biological principle of complex organisms—hampers the development of generic models for predicting meat quality. 

## 2. Stress and Meat Quality

### 2.1. Physiological Mechanisms Underlying the Effects of Stress on Meat Quality

We have known for several decades that slaughter conditions influence meat quality. Research first focused on the production of meat with major defects such as dark cutting (Dark, Firm and Dry (DFD)), and exudative meat (Pale, Soft and Exudative (PSE)) [10]. The former are characterized by a dark color and reduced shelf life and may occur in all major meat species (for review: [11]). Exudative meats are characterized by a light color, low water retention capacity and toughness after cooking. This defect is mainly observed in pigs and poultry but exists also in other species, including cattle [12]. In these early studies, it was rapidly understood that postmortem muscle metabolism plays an important role [13,14]. In the normal process of postmortem biochemical metabolism, after slaughter, biochemical reactions continue, but since blood no longer circulates, glucose and oxygen are not delivered to the muscle. As a result, glycogen stored locally in the muscle is used as an energy source and catabolized anaerobically. Due to the absence of blood circulation, the products of these reactions, in particular hydrogen ions (H^+^), accumulate in the muscle, resulting in a pH decline [15,16]. Contrary to widespread belief, the production of lactate probably does not contribute to the acidification of meat. Lactate production increases when oxygen levels are insufficient. Robergs et al. [15] indicate that in the Lactate Dehydrogenase reaction, for every pyruvate molecule catalyzed to lactate and Nicotinamide Adenine Dinucleotide (NAD+), there is a proton consumed, which makes this reaction function as a buffer. Hence, lactate retards, rather than causes, acidosis. Increased lactate production coincides with cellular acidosis and is a good indirect marker for cell metabolic conditions that induce metabolic acidosis. PSE meats are characterized by a fast pH decline: the low pH during the early postmortem period, while the muscle is still warm, leads to cellular disruption, protein denaturation within the myofibrils and increasing light reflectance from the meat surface and drip loss [12]. DFD meat retains a high ultimate pH caused by a depletion of glycogen stores. Species, breeds, and muscles differ in the likelihood to develop PSE or DFD meat.

It was also understood that the effect of slaughter conditions on muscle metabolism and meat quality could be at least partly explained in terms of animal stress [13,14]. At these early stages of this research topic, stress was often described as the animal’s state when its adaptive capacities were exceeded by the constraints of the environment [17]. Today, many scientists argue that to understand animal stress, we must take into account the emotional state of the animal [18,19,20,21,22] (see Box 1). In the present work, stress is defined as the negative emotional state of the animal in response to a real or imagined threat, associated with a set of behavioral and physiological reactions [23]. Hence, the stress of the animal has a strong subjective component depending on how it interprets the situation it is in. Slaughter includes a series of potentially stressful procedures, starting on the farm and ending with the death of the animal. The procedures may involve food deprivation, gathering and mixing of the animals, transport to the abattoir, lairage and repeated handling. Related to the procedures, some stressors are of physical or physiological origin, such as food deprivation, fatigue or pain. Others are of psychological origin, such as the presence of, often unfamiliar, humans, separation from members of the rearing group and the presence of unfamiliar conspecifics and contexts [24].

Box 1 **Animals and emotions.**

Research provides scientific arguments for the existence of emotions in mammals and various other vertebrates. In humans and nonhuman mammals, the brain contains a network of structures, called the limbic system, which is in charge of the processing of emotions [25,26]. Small, specific lesions in the limbic system modify the expression of emotions, sometimes very specifically, but recent research indicates that the processing of emotions is best described as the output of the integrative functioning of the limbic and other networks, rather than associating specific structures, such as the amygdala, with specific emotions, such as fear [27]. The avian brain contains structures with similar functions as the mammalian limbic system, and behavioral studies show that birds are capable of positive and negative emotions [28,29,30]. Although some controversy exists (e.g., [31]), various scientists indicate that neuroanatomical data and behavioral studies suggest that, like mammals, fish experience various forms of negative emotions [32,33]. For example, lesions of specific parts of the brain affected emotional behavior in goldfish [34], and cortisol levels rise during crowding or handling in fish similarly to mammals [35,36]. Thus, many mammalian brain structures are not present in the fish brain, but other structures may fulfill their functions.

Stress, whether of physical or psychological origin, induces behavioral and physiological changes. With its behavioral stress responses, the animal has the intention to protect itself against the perceived threat. These responses involve defense and avoidance reactions; in the first case, the animal aims to drive the threatening factor away, and in the second, to move itself away from the threat. The animal may also immobilize in order to remain undetected. The physiological stress responses allow the increased vigilance and effort needed for the behavioral responses. They involve increases in heart rate and the secretion of “stress hormones” such as cortisol (corticosterone in birds) and catecholamines (adrenaline and noradrenaline). Preslaughter physiological and behavioral reactions of the animal can have a significant impact on the quality of meat via their effects on muscle energy metabolism. This involves changes in metabolite concentrations and glycogen levels [14,16,37,38]. 

The effects of increased muscular effort and hormonal status on muscle metabolism are closely interwoven. The activation of the sympathetic branch of the autonomic nervous system leads to higher levels of catecholamines and faster heart rates. Adrenaline particularly appears to play an important role in the determination of meat quality. Both exercise and psychological stress induce the release of adrenaline into the bloodstream [39]. The effects of adrenaline on meat quality depend on the state of exercise of the muscle: adrenaline stimulates muscle glycogen breakdown specifically in the exercising muscle and has little effect when the muscle is at rest [40]. Hence, psychological stress is likely to exacerbate the effects of the increased muscular efforts inherent to the slaughter procedure on muscle metabolism, at least in part due to rising adrenaline levels.

The effects of preslaughter stress reactions on postmortem muscle metabolism can be distinguished into two broad categories. The extent of pH decline depends on muscle glycogen stores before slaughter. Increased physical activity and psychological stress the day or hours before slaughter are energy demanding and may consume glycogen stores in the muscles. As muscle glycogen fuels much of the postmortem pH decline, it can lead to higher ultimate pH and in extreme cases, DFD meat. In cattle, depending on the season, diet and time of day, basal *Longissimus thoracis* (LT) glycogen content is between 80 and 100 µmol/g glucose equivalents of wet tissue [41,42]. Approximately 40 µmol/g glucose equivalents of glycogen are needed to lower the pH of bovine LT from ~7.0 to 5.5, and the effects of glycogen depletion on ultimate pH are clearly visible if the preslaughter glycogen content falls below 55 µmol/g glucose equivalents of glycogen [43]. In pigs, results were similar, but the slope of the relationship was breed-dependent [37]. The amounts of glycogen used depend further on the properties and function of the muscle [37,44,45]. Obviously, food deprivation, commonly practiced during the slaughter period, contributes to the lowering of the muscle glycogen stores.

On the other hand, if stress reactions take place in the minutes preceding slaughter, whole-body and particularly muscle metabolic activity is high at the moment of death. This high metabolic activity will continue after the death of the animal, possibly even during the rigor and postrigor period [46], causing an acceleration in the pH decline during the early postmortem period, while the carcass remains relatively warm due to increased heat production [12,14,16,37]. In extreme cases, this leads to the production of PSE meat, as explained above [12]. 

Even in less extreme cases, variations in muscle postmortem pH and temperature decline explain a significant part of the variability in the technological and sensory qualities of meat products [47,48,49,50,51]. The relationships are well known for pigs, poultry and fish; in beef and lamb, they exist also but are more complex [47,52]. Irrespective of the species, biochemical mechanisms other than energy metabolism intervene in the effects of stress on meat quality, as will be discussed in the second part of this paper. 

### 2.2. Tell Me Who’s Least Stressed, I’ll Tell You Whose Meat Is Best

**Figure 1:**
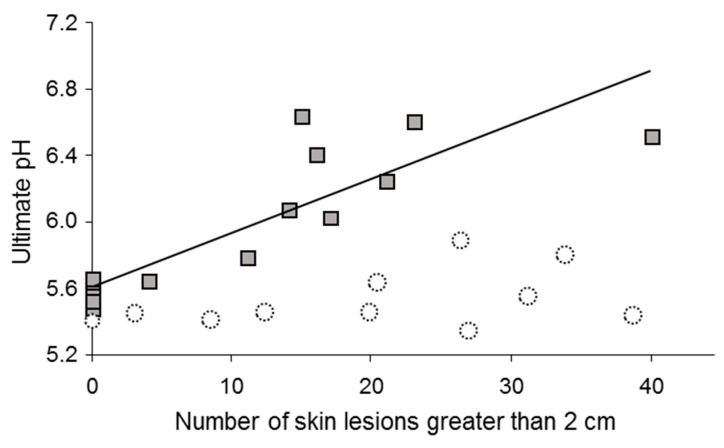
Squares: Piétrain × (Large White × Landrace) pigs. Number of skin lesions, indicative of agonistic interactions during mixed lairage at the abattoir (18 h), were correlated with the pH 24 h postmortem of the *Adductor femoris* muscle (*r* = 0.89; *p* < 0.001). Adapted from [55,61], with permission from INRAE, 2020. Circles represent nonexisting hypothetical data from Hampshire pigs in which fighting is not expected to influence ultimate pH due to their high muscle glycogen content. If all points are combined irrespective of breed, the correlation is no longer significant showing the necessity to take influencing factors into account in the statistical model (see Section 3.1).

**Figure 2:**
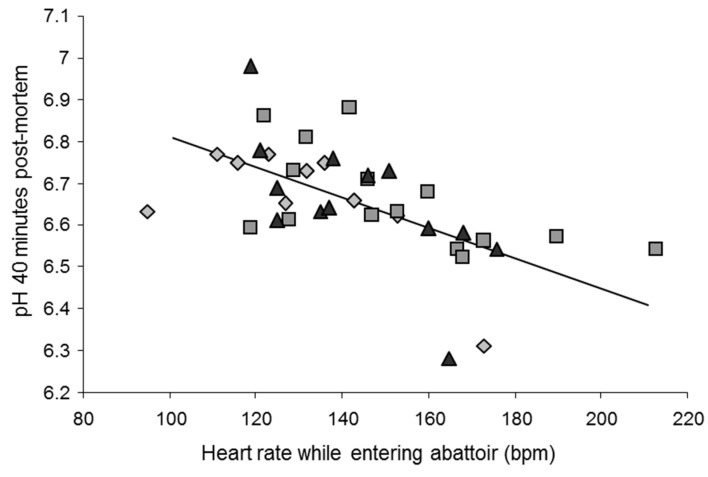
Faster heart rates are associated with faster early postmortem pH decline of the *Semitendinosis* muscle, explaining 34% of the variability between the individuals (*r* = 0.58; *p* = 0.0001). Rhombuses, squares and triangles represent Angus, Blond d’Aquitaine and Limousin young bulls, respectively. Reproduced from [38], with permission from Elsevier, 2020.

In cattle and pigs, certain preslaughter conditions, such as mixing animals or long-term transportation, increase the risk of the production of meat with high ultimate pH at the group level [37]. Studies looking at such effects in detail showed that in pigs, levels of fighting were linearly related to increases in ultimate pH at the individual level (Figure 1; [53,54,55]). In pigs, faster heart rates or higher catecholamine levels before slaughter were correlated with a high rate of early postmortem pH decline and a higher ultimate pH, respectively, impacting meat color and water-holding capacity [37,54,56]. Similar results were found for cattle. In cows [57] and young bulls [38], the higher the heart rates during the minutes preceding slaughter, the faster the decrease in muscle pH during the early postmortem period (Figure 2). Other results show that preslaughter stress not only influences the rate of pH decline but also the sensory qualities of beef. For example, the use of the electric goad during slaughter caused a decrease in sensory quality, including tenderness, assessed by consumers [58]. The effects are proportional to the degree of stress, as preslaughter behavioral and physiological stress indicators and beef tenderness or juiciness showed negative correlations [59,60].

There are only few reports on the relationships between preslaughter stress and meat quality in lambs. Compared to transportation on good-quality roads, transporting lambs on secondary roads caused more pronounced increases in plasma cortisol levels and heart rate, higher ultimate pH and redder or darker meats [62,63]. Longer transport durations also produced tougher and redder or darker meats [64,65]. Physical effort just before slaughter may lead to DFD rather than PSE characteristics if the effects of glycogen depletion outweigh those of the acceleration of metabolism. This was observed in a study on lambs, which were subjected just before slaughter to physical exercise resulting in muscular fatigue and heavy panting as well as the production of meat with DFD characteristics, including high ultimate pH, dark color and greater tenderness and juiciness [66].

In poultry, transport, lairage and shackling are major causes of stress. With increasing transport duration, early postmortem pH decline is increasingly slower, whereas ultimate pH is higher [45,67,68]. During transport, the potential stressors include vibrations, truck movements, impacts, social disturbances and noise [69]. Thermal stress depends on the level and variations in temperatures and air humidity and is one of the most important stresses during transport and lairage; it may reduce muscle glycogen content and increase ultimate pH [69,70,71,72,73]. 

**Figure 3:**
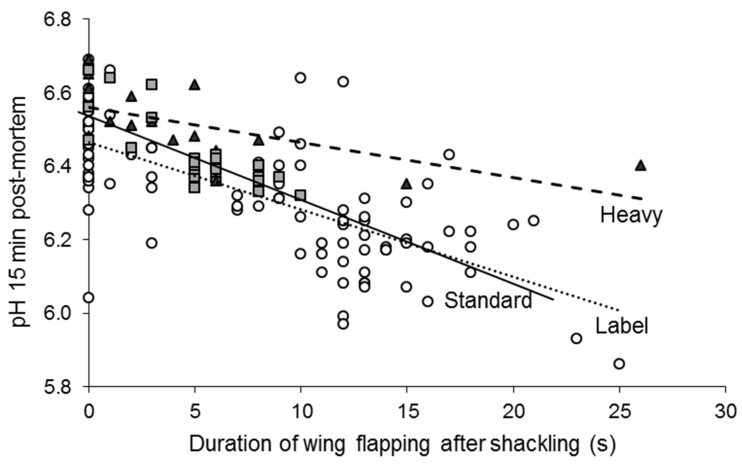
Duration of wing flapping significantly hastens the rate of early postmortem pH decline of fillets. Squares, circles, and triangles indicate broilers of a Standard (*r* = 0.80; *p* < 0.001), Label (*r* = 0.71; *p* < 0.001) and Heavy line (*r* = 0.66; *p* < 0.001), respectively. Slopes do not differ significantly (ANCOVA on pH 15 min postmortem; interaction line x duration of wing flapping: *p* = 0.51). Data from [71].

Shackling of fowl is a source of pain and fear, causing vocalizations and wing flapping, which can lead to injury [74,75,76,77]. The longer the delay between shackling and stunning, the higher the plasma levels of corticosterone, glucose and lactate, and the lower the remaining muscle glycogen stores, indicating greater stress [71,75,77]. The hanging position induced various degrees of wing flapping, which accelerated the early postmortem pH decline proportionally, explaining up to 64% of the variability in the early pH of the *pectoralis* muscle between individuals (Figure 3; [70,71]). Other studies have shown that the redness of fillets of chickens and turkeys increased with longer durations of hanging and of struggling during hanging, respectively [72,78], with the most pronounced effects in slow-growing strains [71].

In fish, regrouping, transport and waiting conditions (duration, density of fish and water quality) and the extraction of fish from their aquatic environment are major causes of stress before slaughter. As with the species discussed above, the physiological reactions and muscular activity associated with stress influence the rate of postmortem pH decline [79,80,81]. For example, in salmon and trout, longer transport durations or increased physical effort before slaughter accelerated the early postmortem pH decline and the installation of *rigor mortis* compared to controls [82,83,84]. Slaughter conditions can also influence the ultimate pH in fish flesh. Increasing the density of salmon 24 h before slaughter was associated with a decrease in muscle glycogen and a higher pH 5 and 14 days after slaughter [85]. Adverse slaughter conditions negatively further affected the flesh, with reduced lightness and yellowness and a softer texture [50,86]. Carp and trout anesthetized with CO_2_, which causes extensive physical struggling and stress, have poorer sensory traits than controls slaughtered by percussion, which causes immediate unconsciousness [23,79]. However, the negative effects of stress on pH or sensory quality traits can disappear 8 to 14 days postmortem [51,85].

### 2.3. Predicting Stress Reactions Is Predicting Meat Quality

**Figure 4:**
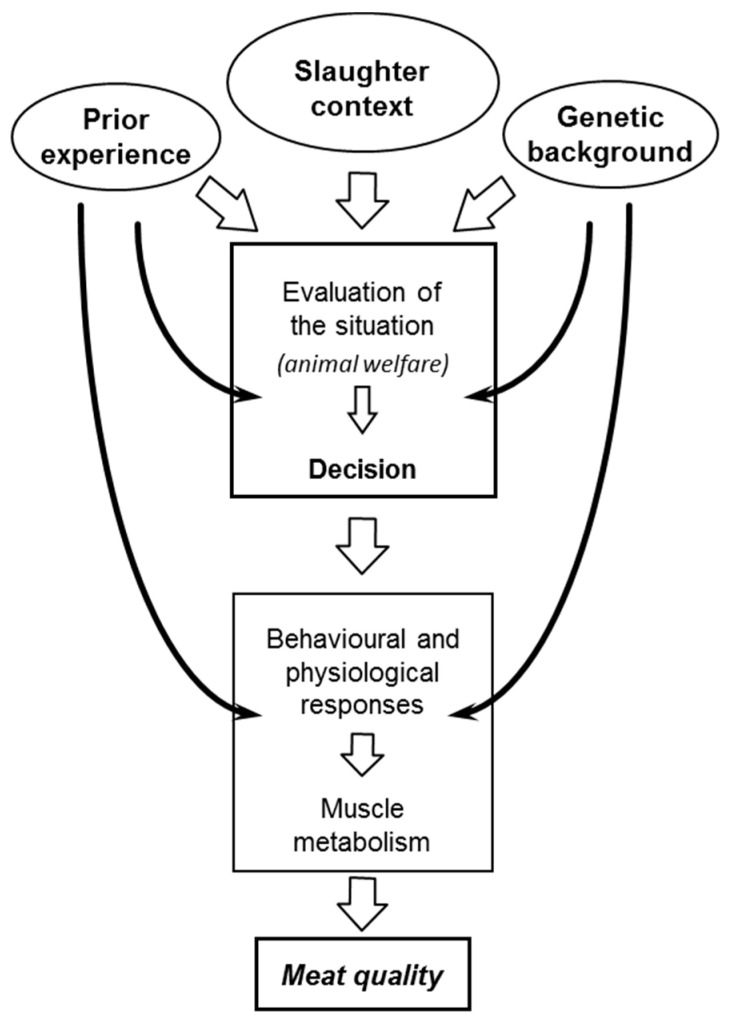
The way the animal evaluates its situation in slaughter (or other) contexts depends on the context itself and the genetic background (e.g., certain breeds or genetic types may be more reactive) and prior experiences of the animal (e.g., the experience an animal has with a certain situation influences the way it evaluates it). The context refers to the physiological state of the animal (e.g., fatigue, hunger, level of arousal, estrus) and its environment (e.g., presence of fear-inducing aspects). The way the animal evaluates its situation is related to its welfare state; the way it reacts to the preslaughter situation influences the qualities of its future meat. The effects of stress on meat quality also depend on the genetic and rearing background of the animal. Reproduced from [87], with permission from Elsevier, 2020.

Animals show a degree of consistency in the way they react to stressors, referred to as the animal’s stress reactivity [37,38]. Stress reactivity is considered high if an animal feels easily threatened and presents pronounced behavioral (whether overt or not) and/or physiological reactions. The way the animal evaluates a situation depends on factors related to the animal: (i) genetic background, which is stable; (ii) earlier experiences, which evolve over the longer term; and (iii) physiological status of the moment, which may change in the short term (Figure 4). As stress reactions to the slaughter context influence meat quality, stress reactivity is a characteristic of the animal that influences its potential for the production of meat of high quality.

By testing each animal in different situations before slaughter, each individual can be characterized for its reactivity to specific situations, relevant to the slaughter context. The consistency in stress reactivity allows identifying animals likely to be more stressed at slaughter than their counterparts. This increases our understanding of the precise causes of stress during the slaughter period. 

**Figure 5:**
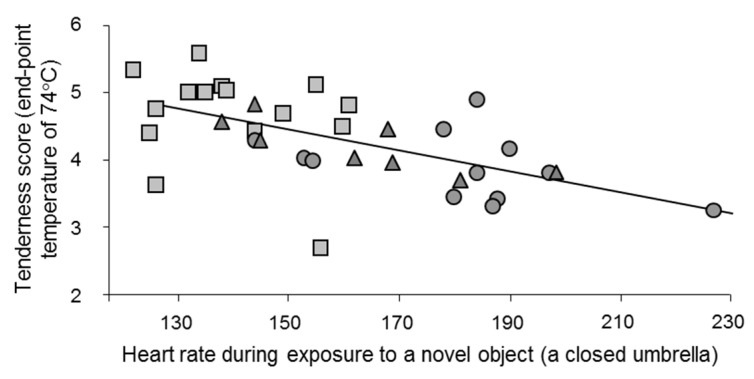
The faster the heart rate response of young bulls of different breeds when exposed to a novel object (a closed umbrella) during a test during the rearing period, the less tender their meat (*r* = −0.59; *p* = 0.01). The tenderness scale goes from 0 (tough) to 10 (tender). Squares, circles and triangles represent Angus, Blonde d’Aquitaine and Limousine bulls, respectively. A faster heart response is indicative of increased fear reactions in unfamiliar situations. Heart rate data are from [38] and meat quality data from [88,89], who worked on the same animals. Adapted from [61], with permission from INRAE, 2020.

Various studies compared the stress reactivity profile, measured during rearing, and stress reactions at the slaughter of individuals of various species. It was found that pigs that were less likely to approach humans (more fearful and/or less attracted) during a test were more reactive to slaughter, as indicated by a faster postmortem muscle metabolism. This suggests that the presence of humans was a significant factor in stress reactions during the preslaughter period [37]. Similar results exist in cattle. Normand cows that showed more stress reactions in an unfamiliar situation during a test conducted three weeks before slaughter had higher heart rates at the start of transport, were more difficult to introduce into the abattoir and had higher catecholamine levels at slaughter. Postmortem, their *Semitendinosus* muscle (fast glycolytic) presented a rapid pH decline and higher temperature, indicative of a faster metabolism [57]. In an experiment on young bulls, heart rate responses in the presence of an unfamiliar object were measured three weeks before slaughter. Following slaughter, the LT muscle of the bulls with a faster heart rate than their conspecifics during the exposure to the object presented a rapid early postmortem pH decline and was tougher during sensory testing (Figure 5; [38,88,89]). In the above experiments, the cows and bulls that were more reactive to unfamiliar situations during the tests were probably more stressed by the unfamiliar slaughter situation, leading to faster ante- and postmortem muscle metabolism [24]. As indicated above, the mechanisms underlying the effect of preslaughter stress on beef toughness are unknown. Preslaughter stress causes calcium release, which not only influences postmortem glycolysis as described above but other pathways such as calpain-mediated proteolysis, autophagy, apoptosis onset and other central processes in meat tenderization. These questions need further investigation and are beyond the scope of this paper.

Sometimes, apparently unrelated behavioral tendencies are correlated. In pigs, for unknown reasons, the tendency to explore a novel object and aggressiveness toward other pigs when challenged are associated [90,91,92]. In agreement with this, pigs that explored an unfamiliar object longer during a test fought more when mixed with other pigs during the slaughter period. As a result, the meat of these pigs had a higher ultimate pH [37,53]. In the above studies, the reactivity to stress measured during rearing could explain up to 70% of the variability observed on the ultimate pH and the color (L *, a *, b *) of the meats [37,53].

Sheep are also consistent in their stress reactivity [22,93]. However, although their responses in stress reactivity tests conducted during the rearing period, especially in social isolation tests, predict their reactions to slaughter, the effects were minor compared to the other species [94]. Poultry show consistency in stress reactivity [95], but there are currently no reports on correlations between reactions to tests during the rearing period in relation to reactions during the preslaughter or slaughter periods. For example, the tonic immobility score established for each animal one week before slaughter was not correlated with reactions to shackling [70]. 

As indicated above, earlier experience modulates stress reactions, including at slaughter, and consequently influences meat quality (Figure 4). Various experiments illustrate this. For example, prior to slaughter, outdoor (extensive conditions) and indoor (conventional limited space conditions) reared pigs were mixed. Compared to outdoor pigs, indoor reared pigs had more skin lesions, suggesting that they had fought more. This resulted in lower muscle glycogen content at the time of slaughter and higher ultimate pH of the meat [94]. Similar results were reported by Barton-Gade [96], suggesting that mixing leads to more aggression in conventionally than outdoor reared pigs, probably because pigs reared in a confined space do not learn to withdraw during conflicts. As indicated above, human presence may be another cause of stress at slaughter. The effect of human presence depends on the earlier experience of the animal with humans [97]. Pigs negatively handled on-farm had lower glycogen levels just before slaughter [53,98]. A faster early postmortem pH decline was observed in the muscles of calves produced by farmers with a negative attitude [99]. Positively handled calves were easier to handle, had lower heart rates during loading and had a higher muscle glycogen content at slaughter compared to controls [99]. Thus, a negative experience with humans during rearing may increase fear of humans and therefore stress reactions at slaughter, resulting in faster muscle glycogen catabolism before and after slaughter, whereas positive experiences produce the opposite effects. 

Under certain conditions, the lack of fear of humans may be counterproductive: pigs that were less fearful of humans were more difficult to drive, and these pigs received more negative interventions from abattoir personnel [53,100]. Probably for the same reasons, in one study, bulls reared by farmers with a positive attitude were more difficult to load [101]. Hence, reduced fear of humans has positive consequences on meat quality, particularly if the equipment for loading and the corridors for moving animals are well conceived, and little human intervention is needed [102]. If moving the animals relies on human interventions, very low fear of humans may make driving the animals more difficult.

Several experiments illustrate the effect of genetic background on stress reactivity (Figure 4). During a human exposure test, Duroc pigs touched the human significantly more often than Large Whites. Resulting from their greater activity levels, Durocs also had faster heart rates during the test. This breed difference was specific for the motivation to touch a human because these same pigs did not differ behaviorally or physiologically in tests exposing them to an unfamiliar object [37]. Using a human exposure test and a surprise test (sudden opening of an umbrella), young Angus, Blond d’Aquitaine and Limousin bulls differed in more than ten behaviors. Thus, bulls of the most reactive breed, Blond d’Aquitaine, showed more startle responses and escape attempts in the surprise test and were more vigilant when exposed to human presence than Angus bulls [38]. Other studies found that beef compared to dairy breeds had a greater flight distance when approached by a human [103], and different beef cattle breeds varied consistently in reactivity to handling and other challenges [104,105,106]. Divergent lines of Leghorn chickens selected for high and low feather pecking showed different increases in plasma cortisol levels when they were restrained [107]. In trout, divergent genetic lines could be obtained based on the rise in plasma cortisol in response to manual restraint, demonstrating the genetic component in their reactivity to emotional stress [50]. 

**Figure 6:**
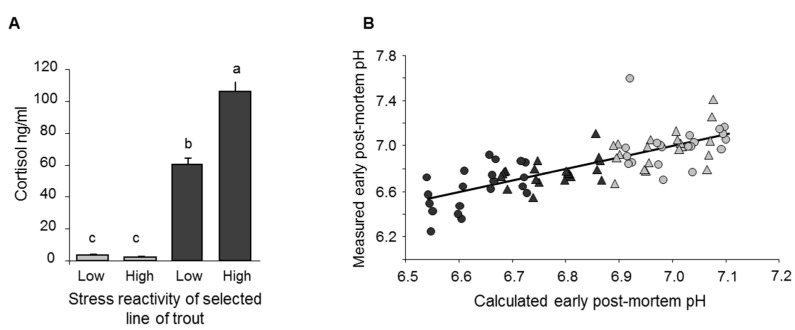
(**A**) Plasma cortisol levels of trout just before slaughter (bars where letters (a–c) differ have significantly different values (*p* < 0.0001)) and (**B**) relationship between calculated and measured pH just after slaughter: pH = 6.8 − 0.003 × slaughter order +0.1 × high reactivity +0.4 × No additional stress at slaughter −0.2 × high reactivity × No additional stress at slaughter. The equation explains 53.3% of the variability between individuals. Dark bars (A) and dark symbols (B) indicate slaughter following additional stress (15 min waiting in a tank with 20 cm depth of water). Triangles and circles indicate selection lines with high and low stress reactivity, respectively. Adapted from [61,81], with permission from INRAE, 2020 and Elsevier, 2020.

Although the breed effects on stress reactions are well established, so far, there are only a few reports on its consequences for stress reactions in abattoirs and meat quality. In an abattoir study, Blond d’Aquitaine bulls were more reactive than Charolais bulls [108]. Several studies comparing physiological stress status between different cattle breeds at slaughter found different urinary catecholamines and cortisol levels, which, in one study, was associated with darker meat [38,109,110,111]. Compared to a fast-growing standard line, broilers of a slow-growing French Label-Rouge line showed greater levels of wing flapping on the shackle line associated with a faster rate of pH decline (see Section 2.1; [70]). When different selection lines of trout based on reactivity to manual restraint were slaughtered with additional stress compared to control conditions, the more reactive line had a more pronounced increase in plasma cortisol levels (Figure 6A; [50]). The muscle pH immediately following slaughter was lower in the group slaughtered with additional stress; however, the effect of stress on pH was less pronounced in the more reactive line (Figure 6B) perhaps due to a limiting factor such as a lower reserve of muscle glycogen due to pronounced stress reactions before slaughter [50]. 

### 2.4. Stress at Slaughter: Lessons to Be Learned 

The studies presented above show that even if animals are slaughtered together, under the same conditions, they react differently to the stressful aspects of slaughter. Part of these differences in reactions is related to differences in their stress reactivity, a measurable characteristic of the animal.

The preslaughter stress status of the animal should be taken into account in statistical models predicting meat quality. Animals differ in stress reactivity, and their stress status will differ even if the slaughter procedure is standardized. Hence, their stress status needs to be measured or slaughter stress has to be very low. In the latter case, results are not necessarily relevant for normal commercial slaughter conditions, where stress levels are generally high. Stress status at slaughter is not the only factor to take into account; muscle, animal gender, age, breed, feeding and rearing/production system, among others, influence meat quality and should be standardized or introduced into the model [2,9,52,112,113,114,115,116,117]. It is essential to take into account all of these factors if we are to succeed in producing pre-hoc statistical models predicting meat quality based on biochemical characteristics of the muscle or other phenotypical features. 

Preslaughter stress reactions have negative economic consequences as they may strongly influence instrumental and sensory meat quality. Consequently, increased stress reactivity of the animal may reduce its potential to produce high meat quality. Efforts of weeks and months of farmers to produce high-quality meat animals may be lost over a few hours if stress is high during slaughter. There are also ethical reasons. As animals are capable of experiencing negative feelings (Box 1), humans have the ethical obligation to avoid stress as much as possible, including at slaughter. To reduce preslaughter stress, the obvious solution is to optimize slaughter conditions using appropriate equipment and employing skilled operators to handle animals [102]. Selecting animals with very low stress reactivity is not a solution; animals with either very high or very low stress reactivity are difficult to handle [38,53,100]. Such a selection may further have inadvertent negative effects on production or quality traits. 

## 3. Multiple Factors Begging Our Attention

### 3.1. Grasping the Erratic Behavior of Correlations: Too Many Uncontrolled Factors! 

In biology, statistical correlations between variables may pop up and disappear like playing cards in the hands of a magician. A correlation may be present in the first repetition of an experiment and be absent or go in the opposite direction in the second repetition. A proportion of correlations is significant by chance, but correlations should not be discarded too easily, even if they seem inconsistent or weak. A significant correlation indicates that a direct or indirect relationship between the variables may exist, but the absence of correlation does not mean the absence of relationships. Correlations may remain undetected if additional influencing factors are ignored; for example, when animals of different breeds are combined in a single analysis and the breed effect is ignored.

As mentioned above, it was found that a given decrease in muscle glycogen affected ultimate pH more strongly in Large White than in Duroc pigs; if the breeds were not considered separately, the overall correlation would be weak or not significant (Figure 5, [37]). Similarly, many Hampshire pigs have high basal muscle glycogen levels due to the presence of the RN-allele in part of the animals of this breed. For these pigs, even following stressful slaughter, ultimate pH remains in the normal range [118], and a correlation between fighting levels and ultimate pH is not expected (see Figure 1). Beyond known major genotypic pathologies, individual variability in expression levels of certain genes, such as 5′-AMP-activated protein kinase subunit gamma-3 (PRKAG3), involved in pathways of physiological stress responses or genetic mutations in promoter regions, can impart variability among individuals in meat quality [119].

**Figure 7:**
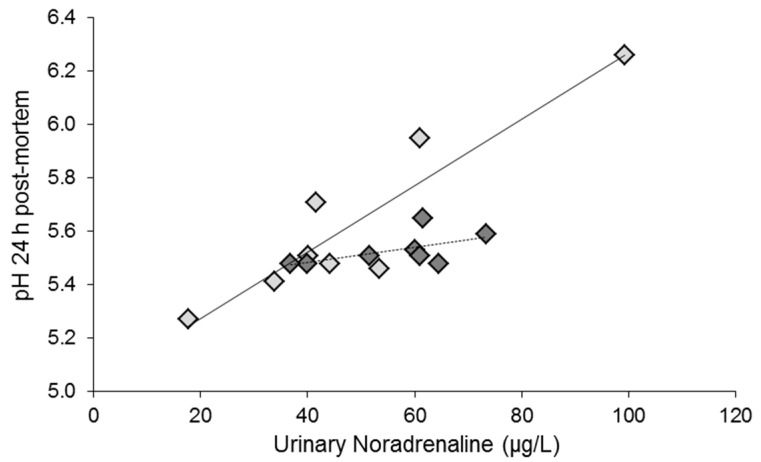
The slopes of correlations between the noradrenaline (NA) content of urine obtained just after slaughter and the pH of the *Longissimus lumborum* measured 24 h postmortem depended on the slaughter method. Pigs had been slaughtered either following mixing and transport to the abattoir the day before slaughter (light-gray symbols; pH = 5.02 + 0.00001 × NA; *r* = 0.92; *p* = 0.001) or without mixing, immediately following transport (dark-gray symbols; pH = 5.37 + 0.000003 × NA; *r* = 0.57; *p* = 0.14). The slopes differ *p* = 0.004 (ANCOVA on pH; NA content × slaughter method: *p* = 0.02). Data from [120] and adapted from [61], with permission from INRAE, 2020. Urinary noradrenaline reflects the amount of noradrenaline secreted into the blood over several tens of minutes preceding the sample collection [122].

Preslaughter stress status may also alter relationships between variables. As an example, Kwasiborski et al. [120] found that the slaughter conditions influenced the relationship between catecholamine levels and ultimate pH. In this study, 24 pigs were selected from a larger dataset to obtain a range of values of *Longissimus lumborum* muscle glycogen content at slaughter. Pigs had been slaughtered either following mixing and transport to the abattoir the day before slaughter (longer-term stress due to overnight mixed lairage at the abattoir) or without mixing, immediately following transport (shorter-term handling/transport stress immediately preceding slaughter). The slope of the correlation between urinary noradrenaline content and ultimate pH depended on the slaughter method (Figure 7). The steeper relationship for pigs slaughtered following mixed lairage may reflect the combined effects of increased activity of the autonomic nervous system and increased physical effort due to fighting during lairage overnight, as explained above (see Section 2.1; [56]). For another correlation, the slaughter conditions influenced the output variable but not the slope of the relationship. In the pigs selected by [121], early postmortem pH decline was correlated with the expression of HSP72 and the abundance of the HSP72 protein, a large inducible 72 kDa Heat-Shock Protein. The slopes of the linear models were similar, but there was an additional effect of slaughter conditions, with higher pH following slaughter after overnight lairage (see Figure 2, [121]). 

**Figure 8:**
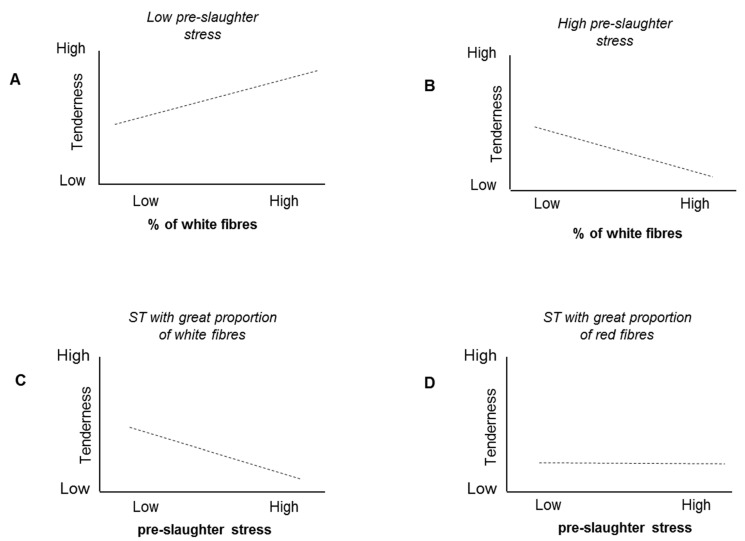
Illustration of the concept that correlations may be reversed if multiple factors are involved in a phenomenon. (**A**) *Semitendinosus* (ST) muscles containing greater proportions of white fibers have greater potential to produce good-quality meat [113], (**B**,**C**) but are also more sensitive to the negative effects of stress; (**B**,**D**) ST muscles containing a greater proportion of oxidative fiber types have less potential to produce good-quality meat but are less sensitive to preslaughter stress. In this example, if we compare low-stress and high-stress slaughter conditions, the correlation between fiber type composition and meat quality is reversed if the negative effects of stress outweigh the positive effects of a high proportion of white fibers (**A**,**B**). The composition of the muscle in terms of fiber type determines whether stress has a measurable impact on meat quality (**C**,**D**). High preslaughter stress will influence the postmortem metabolism of any muscle. Adapted from [61], with permission from INRAE, 2020.

The implication of several factors in a biochemical relationship may even reverse the direction of a correlation, as illustrated by the following, partly conceptual, model. Following slaughter under conditions of minimal stress, the higher the proportion of white fibers (glycolytic type) in the *Semitendinosus* (ST) muscle of young bulls, the tenderer the meat [113]. However, compared to red fibers (oxidative type), white fibers are equipped with enzymes that have a greater capacity to accelerate their activity under stressful conditions [123,124,125], potentially decreasing meat quality, such as juiciness and tenderness, as indicated above [14,16,59]). Thus, ST muscles containing greater proportions of white fibers have greater potential to produce tender meat ([113], Figure 8A) but are also more sensitive to the negative effects of stress on meat quality (Figure 8B,C). ST muscles containing a greater proportion of oxidative fiber types show the opposite tendency (Figure 8B,D). Based on our knowledge of the effects of stress on various meat quality traits, particularly if they contain predominantly white fibers, it is expected that the correlation between fiber type composition and meat quality may be reversed if we compare low-stress and high-stress slaughter conditions. While under low-stress slaughter conditions, the percentage of white fibers and high meat quality are positively correlated (Figure 8A); following slaughter under stressful conditions, if the negative effects of stress outweigh the positive effects of a high proportion of white fibers, this correlation is negative (Figure 8B). Hence, if multiple factors are involved in a biological phenomenon, opposite correlations do not necessarily contradict the reality of an underlying relationship; they may simply indicate that additional biochemical events are involved [4,126]. Examples of opposite correlations are the negative relationship between tenderness and glycolytic profile of the beef LT muscle compared to the positive correlation observed for the ST stated above [113] and the opposite correlations between peroxiredoxin-6 (PRDX6) abundance and color intensity (Chroma) according to breed [3]. The correlations between small heat-shock proteins and tenderness were also opposite in ST and LT [113]. LT and ST muscles differ in contractile and metabolic properties and also in abundances of heat-shock and oxidative stress proteins, known to be involved in beef tenderness determination, which may explain the opposite relationships according to breed and gender [2,4,127,128]. 

Whether their directions change or not, recurrent correlations are of particular interest as they are indicative of relatively direct and probably biologically meaningful relationships. Literature reviews allow their identification across generally independent studies [4,126]. They may also be observed within the same study. Using young bulls of three breeds, Gagaoua et al. [2] studied and built correlation networks among proteins that were associated with beef tenderness in two muscles. The constructed correlation network of protein biomarkers of beef tenderness showed that abundances of PRDX6 and µ-calpain were robustly correlated in the two muscles of the three breeds, suggesting that the functioning of these proteins is closely related [2]. In accordance, an in vitro study using rat pancreas insulin-secreting cells showed that PRDX6 is regulated by calpains [129]. 

**Figure 9:**
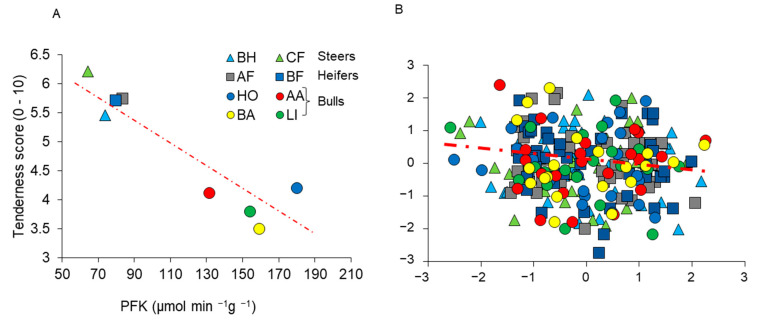
Example of correlation between muscle characteristics and beef quality traits. (**A**) Average values of phosphofructokinase (PFK; glycolytic enzyme) and tenderness score are strongly negatively correlated (*r* = −0.92; *p* < 0.0001) when using average values of different animal types. (**B**) However, when Z-scores are used, removing the effects of animal type, the correlation is nonexistent (*r* = −0.06; NS), indicating that PFK activity was not directly related to tenderness [88]. Animals were steers: 40 Belgian-Blue × Holstein (BH) and 32 Charolais crossbred (CF) reared in the United Kingdom; heifers: 47 Angus × Friesian (AF) and 47 Belgian-Blue × Friesian (BF) reared in Ireland; young bulls: 25 Holstein (HO) reared in Germany, and 24 Angus (AA), 25 Limousin (LI), and 25 Blond d’Aquitaine (BA) reared in France. Higher tenderness scores indicate greater tenderness.

However, not all recurrent correlations reveal causative relationships; lung cancer and yellow fingers may be robustly correlated, but yellow fingers do not cause lung cancer, not even indirectly nor vice versa. In the context of meat quality, Gagaoua et al. [88] showed that in a group of 265 cattle combining eight animal types differing in breed, gender and rearing background, ultimate pH was strongly correlated with Cytochrome c Oxidase, Lactate Dehydrogenase and Myosin Heavy Chain-I. However, within each animal type, these correlations were not observed. Similar inconsistent observations were made for the relationship between the glycolytic enzyme phosphofructokinase (PFK) and sensory tenderness scores (Figure 9). This indicates that the correlations observed across animal types were very indirect, possibly even of genetic origin, and are not very helpful in trying to understand the causes of differences in meat traits between animals [88]. For similar reasons, biochemical characteristics may be correlated across, but not within muscles [117]. 

In summary, correlation analyses may give much insight into the relationships between biological variables. Inconsistencies in correlations do not necessarily invalidate relationships; they often point to the multifactorial character of the biological system that is studied. 

### 3.2. How Many Pathways Lead to a Phenotype? How Many Ways to Make a Tasty Soup?

The changing character of correlations is obviously due to the complex nature of biological systems. To understand this complexity, maybe a useful, albeit imperfect, metaphor is soup (or any other multi-ingredient dish). If the purpose of a biological system is to continue its existence by a correct equilibrium of all its subsystems, the purpose of soup could be defined as “being tasty” due to a correct equilibrium of its ingredients. There are countless tasty soups possible just by changing their ingredients. Likewise, there are countless ways for a biological system to be in homeostasis by adjusting its many processes on various macroscopic and microscopic levels. However biological systems are much more complex, as many processes are dynamic and interwoven, and the state of each endpoint, such as the availability of energy substrates, may be reached through different equilibria. For example, if blood glucose is relatively low, more lipids can be mobilized [41]. A strong interconnection between processes is necessary [4] because their combined output has to be adapted to the situation, aiming for optimal functioning and maximal survival chances. 

To get an insight into complex interactions, the most efficient approach would seem the investigation of the master regulator, as one would contact the traffic control tower to obtain all the information on incoming and departing planes. However, in biological systems, there is not a single control point; exchanges take place on multiple levels. The brain as the supervising organ collects and interprets information on the environment through the senses and the physical and physiological state of the body using various sensors. Certain sensors are located in the brain, relative to, for example, body temperature and blood gas, pH and glucose. The brain further receives much information via the peripheral nervous system from sensors in the body. Stretch receptors provide information on the distension of the lungs; the digestive system; muscle tension and posture; blood pressure; mechanoceptors information on blood pressure or pressure on the skin; chemoreceptors in the carotids on blood gas; other types of chemoreceptors in the skin on injury; thermoreceptors in the skin, liver and muscles on the temperature of the various organs, among others. The brain integrates the different types of information; it adjusts the various physiological processes and makes decisions on the behavior. For instance, during stress, the sympathetic branch of the autonomic nervous system is activated, resulting concomitantly in faster heart rates, faster breathing, faster blood circulation and greater availability of energy substrates. The combined effects increase the availability of oxygen and energy substrates, and the clearance rate of waste products, allowing greater physical effort. 

**Figure 10:**
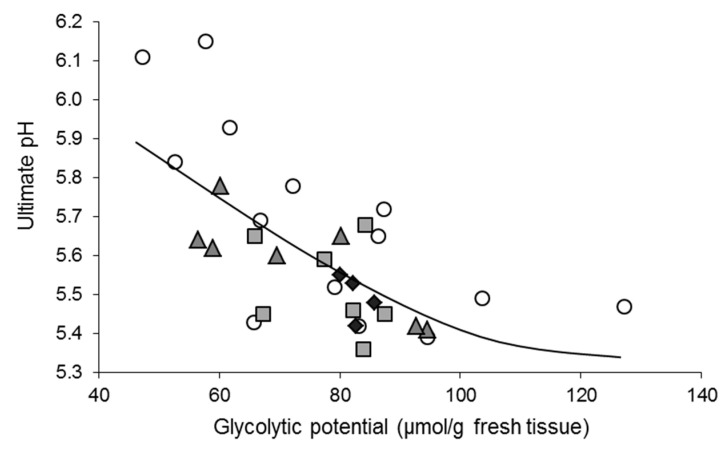
Pig *Semimembranosus* (SM) slaughtered following gas stunning: relationship between the glycolytic potential 45 min postmortem (GP) and pH measured 24 h postmortem. The best logarithmic fit was ultimate pH = 8.40 − 65 × ln (GP), illustrated by the curved line (*r* = 0.69; *p* < 0.0001). The different symbols refer to pigs with different liver GP; circles: 10–39, squares: 40–99, triangles 100–170; rhombuses: 268–289 µmol/g fresh tissue; none of the pigs had values between 170 and 268 µmol/g. GP in muscle and liver was calculated from lactate, glucose and glycogen content, following [138]. The results illustrate that with GP below 80 µmol/g fresh tissue, ultimate pH rises. They also show that some of the pigs with low muscle glycogen had liver GP contents over 100 µmol/g. Hence, although liver glycogenolysis helps to replenish glucose, the remaining liver glycogen contents did not match SM muscle contents. This may be due to a combination of factors; for instance, resting liver glycogen contents may have varied, the rate of glycogenolysis may depend on additional factors, such as stress, or there may be a delay between need and actual glycogenolysis. The figure illustrates the heterogeneity of physiological adaptive responses. Adapted from [139], with permission from ADIV, 2020.

Although the different organs are controlled by the central nervous system, they also interact directly via different means. They exchange metabolites; for instance, lactate produced by the muscles is converted to glucose by the liver, and amino acids produced by the kidneys and liver are consumed by the muscle [130,131]. Organs are further often influenced by the same hormones, but the exact effects differ. For example, in the muscle, insulin allows glucose uptake, allowing the muscle to be active. In adipose tissue, insulin also favors glucose uptake but blocks lipolysis, thus stimulating energy storage. Cortisol, in contrast, facilitates lipolysis and blocks the action of insulin in adipose tissue. Adrenaline stimulates lipolysis and glycogenolysis in the liver and muscle [132,133]. Hence, during stress, when cortisol and adrenaline are high, the combined action of these hormones is that glucose and lipids are made available for increased muscle activity, whereas energy storage is inhibited. The exact balance differs between individuals (Figure 10). Similarly, increased physical effort leads to greater production of CO_2_, part of which dissolves in the blood. If uncontrolled, this would lead to a lowering of plasma pH. However, the lungs and kidneys collaborate to maintain pH within the normal range; the lungs through an essentially centrally driven increase in respiration and the kidneys by increasing acid excretion, driven by local mechanisms in the renal tubules [134,135]. 

On a lower level, the complexity of biological systems is illustrated by interactive molecular processes inside the cell. Using bioinformatics, interactions between pairs of molecules can be compiled into larger interactomes, best represented by interaction maps [4,125,126]. Proteins are the most important building blocks of such networks. These networks show strong functional connections among proteins of the same biochemical pathway; among metabolic enzymes, among antioxidant proteins, among structural proteins and among chaperone proteins such as heat-shock proteins, creating different families of molecules according to their function [4]. Connections exist further between molecules of different families, indicative of interconnectedness among different cellular processes and functions [4]. These interconnections arise when a given pathway produces substrates for another pathway; for example, metabolic activity generates free radicals, thus stimulating antioxidative (scavenging) activity. They may also result from the activity of multitasking proteins. The function of a protein may change due to posttranslational modifications, such as phosphorylation and acetylation. Certain proteins, particularly metabolic enzymes, have moonlighting activities; that is, they can exhibit multiple unrelated functional activities. Hexokinase allows the first step of the glycolytic pathway with the formation of G-6-P from glucose but, in addition, facilitates autophagy in response to glucose deprivation through an independent pathway [136]. Similarly, PRDX6 is a bifunctional protein with glutathione peroxidase activity and phospholipase A2 activity [137]. This characteristic may be relevant for its role in both tenderness and color variation in cattle [4,52,126]. 

From a Darwinian evolutionary point of view, the multiple exchanges among brain functions, organs and intracellular processes allow the versatility and coherence that cells, organs and the whole organism need to constantly adapt and to survive under variable circumstances. For the scientist, it means that the animal we study changes constantly, adapting to the environment and its permanently varying body needs in changing ways and each animal differently, according to specific biological principles. 

### 3.3. Multifactorial Phenotypes: Lessons to Be Learned

**Figure 11:**
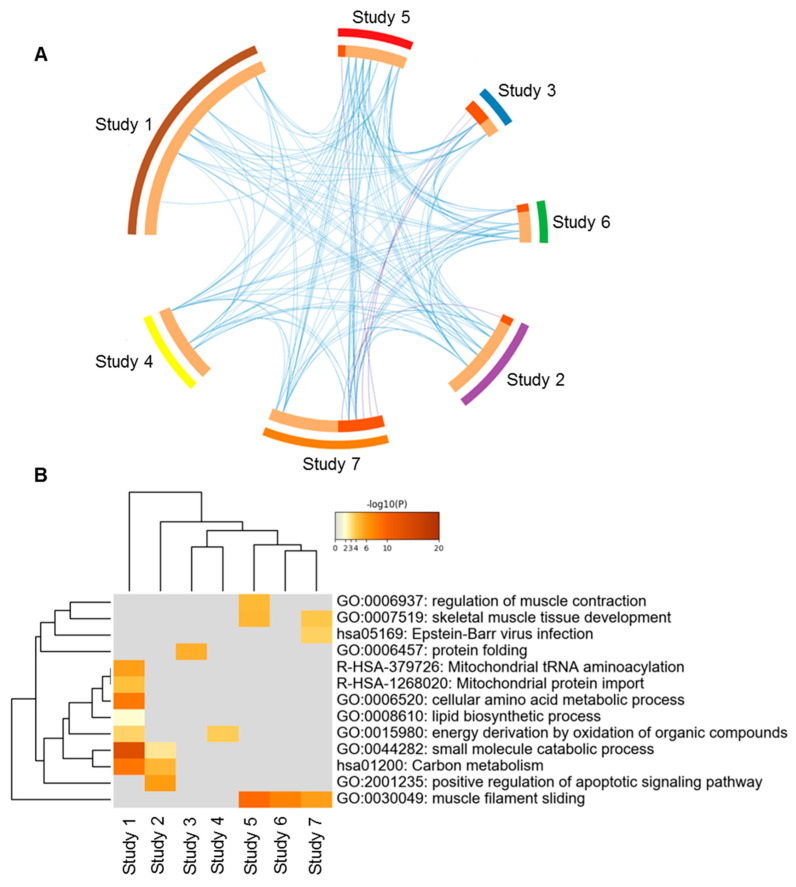
Example of disparity among beef studies on dark-cutting (Dark, Firm and Dry (DFD); ultimate pH > 6.0) *Longissimus thoracis* muscle using proteomics (Gagaoua, unpublished data). The seven selected proteomic studies reported in total 78 proteins, which were compared by means of a Cricos plot for overlap (**A**) and a heat map using the enriched gene ontology pathways for their functions (**B**). In the heat map, colors from gray to brown indicate *p*-values from high to low, and gray cells indicate the lack of significant enrichment. The two analyses based on the proteins or the biological pathways illustrate the little consistency among studies in terms of the identified proteins and pathways involved in the production of DFD meat in contrast to what is known for normal meat on the same muscle, i.e., normal color (Gagaoua et al. [126]) or tenderness (Gagaoua et al. [4]). This inconsistency indicates the existence of other influencing factors that need to be controlled in order to construct accurate and robust models (see Section 4: Conclusion).

Phenotypical traits of animals, muscles or meats are generally described in broad terms, for instance, based on the growth rate, composition, or color. Consequently, groups that appear to be of a similar phenotype are in reality relatively heterogeneous. Thus, meat can be light-colored because of a prevalence of fast-twitch glycolytic muscle fibers, a fast pH decline, low ultimate pH or a combination of these [126,140]. Similarly, both the young age of the animal and high marbling of the muscle may underlie high tenderness [8,141,142]. As mentioned, in the case of muscles and meat, statistical models identify proteins of which the levels differ according to a given phenotypic trait. Subsequently, the biochemical pathways in which these proteins are involved are identified using networks and gene ontology analyses, allowing insight into the functional mechanism underlying the phenotypical trait. Such approaches have identified, for instance, that proteins related to “muscle contraction,” “ATP metabolic process,” “muscle structure development,” “oxidative stress” and “chaperone-mediated protein folding” are interconnected pathways that contribute to the tenderness development of the *Longissimus* muscle in cattle of different ages, breeds and genders [4]. In contrast to tenderness, a meta-analysis on dark-colored meat found very little coherence between studies. Seventy-eight proteins found to be related to dark-cutting meat in seven studies were compared. A Cricos plot (Figure 11A) and functional heat map (Figure 11B) found very few common proteins between the studies, and pathways related to the dark color varied greatly (Gagaoua, unpublished data), which is very different from what is observed for meat with normal color [126]. These results illustrate that more detailed knowledge of the molecular mechanisms involved in the determination of meat quality traits would allow a more precise categorization of different phenotypes [1,143].

Such integromics meta-analyses are essential because they give insight into the biological pathways involved in meat quality traits [4,126]. However, despite our current knowledge, even when studying a single muscle of a given animal type with a known preslaughter stress status, currently, we are unable to estimate future meat quality using the relative amounts of the relevant proteins. There are several reasons for this. As indicated above, different underlying causes may lead to the same phenotypical trait, and each underlying cause would need its own model. Furthermore, a phenotypical trait is the output of the combined interactive collaborations of many proteins. Interactions between proteins may be complex and nonlinear, needing higher-order statistical models to be described adequately [144]. As an example, the Ras-Raf-MEK-ERK pathway is essential for many cellular processes, including apoptosis, and its activity shows an oscillatory pattern. The reason is that activated ERK simultaneously triggers a positive and separate negative feedback loop, which both influence Grb2-SOS activity in opposite ways [145]. Finally, it is important to distinguish between the different isoforms (proteoforms) of each protein [4,120,126]. Particularly, protein phosphorylation is an important cellular regulatory mechanism, as many enzymes and receptors are activated or deactivated by phosphorylation and dephosphorylation events by means of approximately 700 identified protein kinases and phosphatases [146]. Computational network modeling techniques developed in medicine for the identification of biomarkers of pathologies in complex systems [147,148,149] may be relevant for meat science. For example, recent modeling techniques allow describing and predicting dynamic changes in nonlinear complex small-scale systems at the intracellular level or cell–cell interactions [144]. They may have an interest in the modeling of meat quality traits at the cellular and molecular level. 

In summary, many interactive cell processes govern various aspects of meat quality and these are increasingly well known. The creation of pre-hoc predictive or explanatory statistical models remains difficult because of the dynamic and interactive nature of the processes that take place in the cell, the organs and the whole organism, as well as the very large number of parameters involved, only partly described above. In addition, phenotypical similarity does not necessarily imply a similarity of the underlying causes. Results may benefit from modern computational modeling techniques to improve the identification of biomarkers as well as the understanding of small-scale dynamic nonlinear systems.

## 4. Conclusions

The above observations and reflections show that to develop pre-hoc predictive models for meat quality traits, we must refine our approaches and pay attention to details. First, submodels should be established for a given muscle of a given type of animal, referring to breed, age, gender, rearing/feeding system and any other influencing factors. Although the latter factors are often taken into account, another major factor, preslaughter stress status, is generally ignored. Behavioral and physiological stress responses have major impacts, mostly negative, on meat quality traits in various species. Hence, to develop robust predictive models, preslaughter stress must be controlled, which is difficult, or measured. 

Another ignored aspect is that different underlying causes may lead to similar phenotypes. The best initial results will be obtained for models dealing with a phenotype with homogenous underlying causes. Obviously, there must be sufficient variation between animals or cuts if statistical relationships are to be considered. Proteins used as predictors should be identified according to their isoform using appropriate tools. Next, submodels showing similar relationships between phenotypical traits and proteins may be combined into larger models using interactomics. Further, in vitro and in vivo laboratory studies on protein functioning, bioinformatics, data-mining and integromics meta-analyses are paramount for the functional interpretation of the models. Another important aspect is the complex and nonlinear relationships between protein levels and biochemical events. Therefore, such models can only explain part of the variability between individuals. Generic models using biochemical mechanisms underlying meat quality are not expected to be found; rather, a range of models to predict specific phenotypes, taking into account muscle/animal characteristics and preslaughter stress status simultaneously, are used. Other omics approaches such as genomics, transcriptomics and metabolomics may also yield interesting results to produce other, including multiomics, models or help interpreting models based on proteomics.
